# OSR1 disruption contributes to uterine factor infertility via impaired Müllerian duct development and endometrial receptivity

**DOI:** 10.1172/JCI161701

**Published:** 2023-12-01

**Authors:** Adriana Lofrano-Porto, Sidney Alcântara Pereira, Andrew Dauber, Jordana C.B. Bloom, Audrey N. Fontes, Naomi Asimow, Olívia Laquis de Moraes, Petra Ariadne T. Araujo, Ana Paula Abreu, Michael H. Guo, Silviene F. De Oliveira, Han Liu, Charles Lee, Wendy Kuohung, Michella S. Coelho, Rona S. Carroll, Rulang Jiang, Ursula B. Kaiser

**Affiliations:** 1 Molecular Pharmacology Laboratory (FARMOL), Faculty of Health Sciences, University of Brasilia, Brasilia-DF, Brazil.; 2 Section of Endocrinology, Gonadal and Adrenal Diseases Clinics, University Hospital of Brasilia, Brasilia-DF, Brazil.; 3 Division of Endocrinology, Diabetes and Hypertension, Brigham and Women’s Hospital and Harvard Medical School, Boston, Massachusetts, USA.; 4 Division of Endocrinology, Children’s National Hospital, Washington, DC, USA.; 5 Department of Pediatrics, George Washington School of Medicine and Health Sciences, Washington, DC, USA.; 6 Whitehead Institute for Biomedical Research, Cambridge, Massachusetts, USA.; 7 Division of Endocrinology, Boston Children’s Hospital, Boston, Massachusetts, USA.; 8 Program in Medical and Population Genetics, Broad Institute, Cambridge, Massachusetts, USA.; 9 Department of Genetics, Harvard Medical School, Boston, Massachusetts, USA.; 10 Department of Genetics and Morphology, Institute of Biology, University of Brasilia, Brasilia-DF, Brazil.; 11 Jackson Laboratory for Genomic Medicine, Farmington, Connecticut, USA.; 12 Division of Developmental Biology, Cincinnati Children’s Hospital Medical Center, Cincinnati, Ohio, USA.; 13 Department of Obstetrics and Gynecology, Boston University Chobanian & Avedisian School of Medicine, Boston, Massachusetts, USA.; 14 Department of Pediatrics, University of Cincinnati College of Medicine, Cincinnati, Ohio, USA.

**Keywords:** Development, Reproductive Biology, Embryonic development, Genetic diseases, Obstetrics/gynecology

## Abstract

Three sisters, born from consanguineous parents, manifested a unique Müllerian anomaly characterized by uterine hypoplasia with thin estrogen-unresponsive endometrium and primary amenorrhea, but with spontaneous tubal pregnancies. Through whole-exome sequencing followed by comprehensive genetic analysis, a missense variant was identified in the *OSR1* gene. We therefore investigated *OSR1*/OSR1 expression in postpubertal human uteri, and the prenatal and postnatal expression pattern of *Osr1/*Osr1 in murine developing Müllerian ducts (MDs) and endometrium, respectively. We then investigated whether Osr1 deletion would affect MD development, using WT and genetically engineered mice. Human uterine *OSR1*/OSR1 expression was found primarily in the endometrium. Mouse Osr1 was expressed prenatally in MDs and Wolffian ducts (WDs), from rostral to caudal segments, in E13.5 embryos. MDs and WDs were absent on the left side and MDs were rostrally truncated on the right side of E13.5 *Osr1*^–/–^ embryos. Postnatally, *Osr1* was expressed in mouse uteri throughout their lifespan, peaking at postnatal days 14 and 28. Osr1 protein was present primarily in uterine luminal and glandular epithelial cells and in the epithelial cells of mouse oviducts. Through this translational approach, we demonstrated that OSR1 in humans and mice is important for MD development and endometrial receptivity and may be implicated in uterine factor infertility.

## Graphical abstract

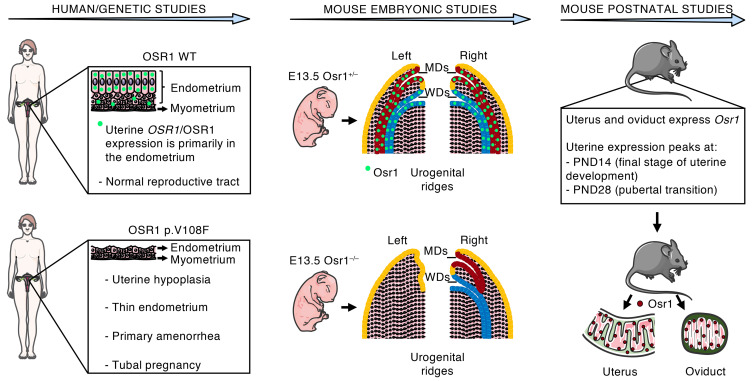

## Introduction

The Müllerian ducts (MD) are paired embryonic structures that arise from the mesoderm-derived invaginations of the coelomic epithelium into the mesonephros and ultimately develop into segments of the female reproductive tract (FRT) — the oviducts, uterus, cervix, and upper part of the vagina (1, 2). Postnatally, MD derivatives function as the site of fertilization in many vertebrates, including humans and mice, and play fundamental roles in oocyte transport and nutrition, as well as in embryo development, thus determining reproductive success (3, 4).

Müllerian anomalies comprise a heterogeneous group of developmental defects that affect FRT anatomy and function, ranging from minor uterine abnormalities, such as septate and bicornuate uteri, to congenital absence of the uterus and vagina (1, 5). One of the most severe conditions in the spectrum of Müllerian anomalies in humans is Mayer-Rokitansky-Küster-Hauser syndrome (MRKH), or uterovaginal agenesis, among the most common causes of primary amenorrhea. Approximately 1 in 4,500 live births have MRKH, representing 15% of women with primary amenorrhea. Rare cases of familial clustering and the association with other congenital defects, such as renal or skeletal malformations, have directed the search for a genetic basis of the disease (5, 6).

We studied a family in which 3 sisters, born from consanguineous parents, presented with a unique Müllerian anomaly characterized by uterine hypoplasia with a thin endometrium unresponsive to sex steroids and primary amenorrhea, but spontaneous tubal pregnancies. Our hypothesis was that a recessive genetic factor may have caused this familial phenotype, leading to disruption of MD development and patterning and a resultant reproductive tract developmental defect characterized by estrogen-resistant endometrial hypoplasia and impaired uterine embryo implantation. Through whole-exome sequencing (WES) followed by comprehensive genetic analysis, odd-skipped related 1 (*OSR1*) emerged as a candidate gene. *OSR1* is the human homolog of the drosophila odd gene, which encodes a transcription factor expressed early during embryonic development in mice, beginning in the nascent intermediate mesoderm, and subsequently expressed in multiple mesenchymal derivatives in mice, including coelomic epithelium and the urogenital ridges where MDs originate (7, 8). We therefore aimed to investigate the *OSR1*/OSR1 expression in the developed human uterus, and to examine the prenatal and postnatal expression pattern of *Osr1/*Osr1 in developing MDs and in the endometrium, respectively, and whether its deletion would affect MD development, using WT and genetically engineered mice, including *Osr1*^–/–^ (9), *Osr1^FLAG/+^*, and *Osr1-LacZ* (8) mice. Through this translational approach, we demonstrate that *OSR1* is a gene important for MD and endometrial development that may contribute to uterine factor infertility in women.

## Results

### Human studies

#### Case report.

**Figure 1:**
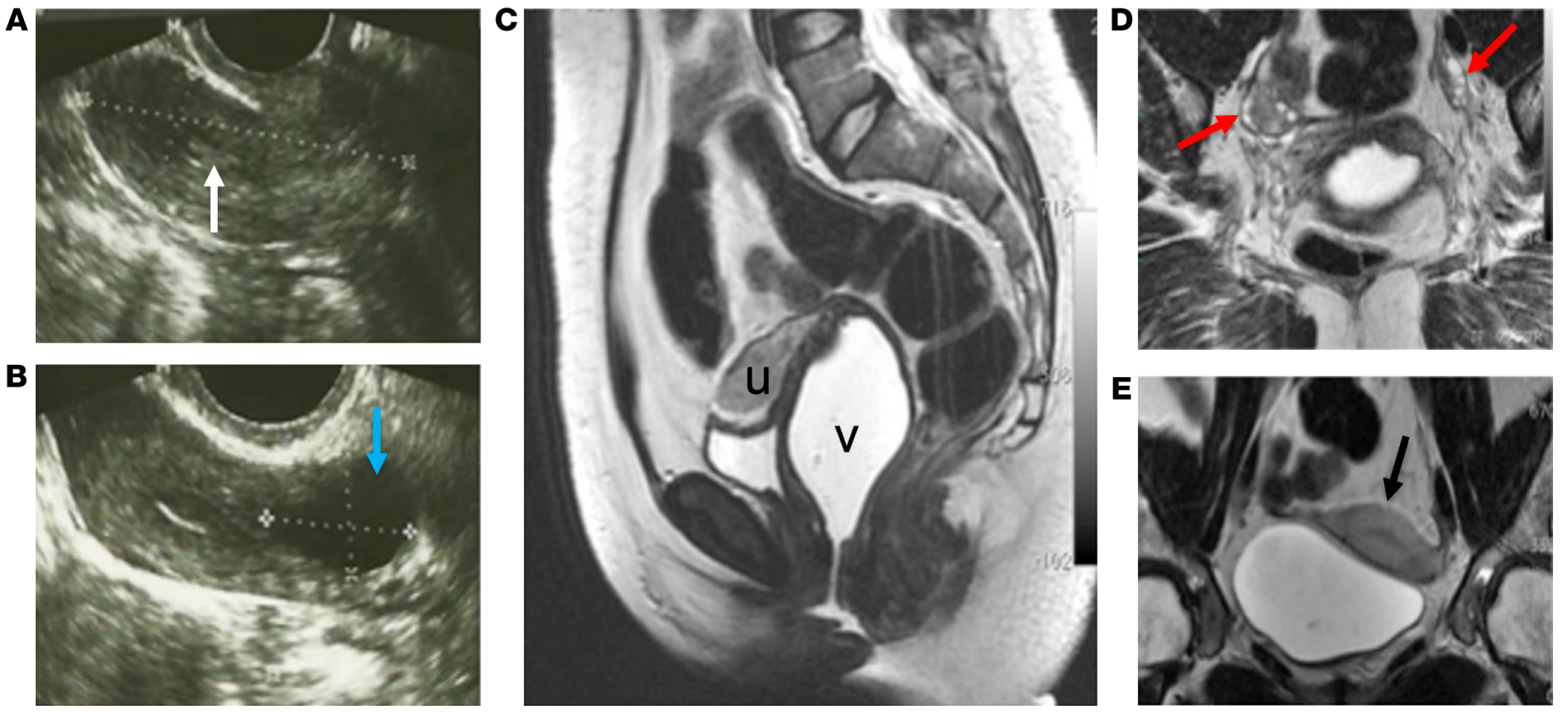
FRT imaging features of a 29-year-old woman (proband) with primary amenorrhea and a developmental anomaly of the reproductive tract. (**A** and **B**) Transvaginal ultrasonographic imaging showing (**A**) a hypoplastic uterus (dotted line) with a very thin endometrium (white arrow), and (**B**) a left cornual cystic lesion (blue arrow); (**C**–**E**) Sagittal (**C**), oblique coronal (**D**), and coronal (**E**) T2-weighted fast-recovery fast spin-echo (FRFSE) MRI showing pelvic sections with vaginal distention using aqueous gel, demonstrating: (**C**) widening of the upper 2 thirds of the vagina (v) and an anteverted hypoplastic uterus (u), (**D**) normal appearing ovaries (red arrows), and (**E**) a hypoplastic uterus with thin endometrium (black arrow). The MRI of the proband was performed while off of hormonal treatment for more than 1 year. No information about cycle phase was available since the patient was amenorrheic.

The proband was a gravida zero 29-year-old Brazilian female who reported primary amenorrhea and otherwise complete development of secondary sexual characteristics by the age of 12. She started cyclic combined oral contraceptives at age 22 and had only 3 episodes of minimal spotting over 2 years despite taking a week of placebo pills every month. General physical and pelvic examinations were unremarkable. Her weight was 48.9 kg and height was 149.7 cm, with a normal body mass index of 21.8, and karyotype was normal female 46,XX. A progesterone challenge test failed to induce menstrual bleeding. Transvaginal ultrasonography (TVUS) showed a hypoplastic uterus of 13 cm^3^ (the reference range for nulliparous women is 28–65 cm^3^) with a thin endometrium of 1 mm (the normal range of endometrial thickness for premenopausal adult women is 4.0–8.0 mm and 8.0–12.0 mm in the follicular and luteal phases, respectively), and a cystic lesion (18 mm) in the left cornual region (Figure 1, A and B). Weekly hormone assays and TVUS scans showed normal appearing ovaries (with follicles up to 10 mm), with normal folliculogenesis, ovulation, and corpus luteum formation, but an impaired endometrial proliferative response (Supplemental Table 1; supplemental material available online with this article; https://doi.org/10.1172/JCI161701DS1). To further evaluate endometrial estrogen responsivity, estrogen supplementation was administered orally (4 mg/day; Estrofem, Medley). A pelvic ultrasound was performed after 9 weeks of use and showed no change in endometrial thickness. Renal ultrasonography showed no abnormalities. Dual-energy X-ray absorptiometry (DEXA) showed a normal bone density Z-score, further supporting normal estrogen action on peripheral tissues. Pelvic MRI confirmed the TVUS findings and, in addition, revealed widening of the upper third of the vagina (Figure 1, C–E). At age 33 years, a left tubal pregnancy was documented by ultrasonographic visualization of an adnexal mass and elevated serum β-hCG levels. This ectopic pregnancy was managed conservatively with inpatient observation; she was discharged after hCG levels had declined to the nonpregnant reference range.

Family history was notable for parental consanguinity (first degree cousins) and 2 sisters who also had primary amenorrhea with normal secondary sexual characteristics and hormone levels, despite underdeveloped uteri and thin endometrial thickness. Interestingly, one of these sisters reported a prior ruptured left tubal pregnancy that had been treated surgically at a remote a facility (records not available).

**Figure 2:**
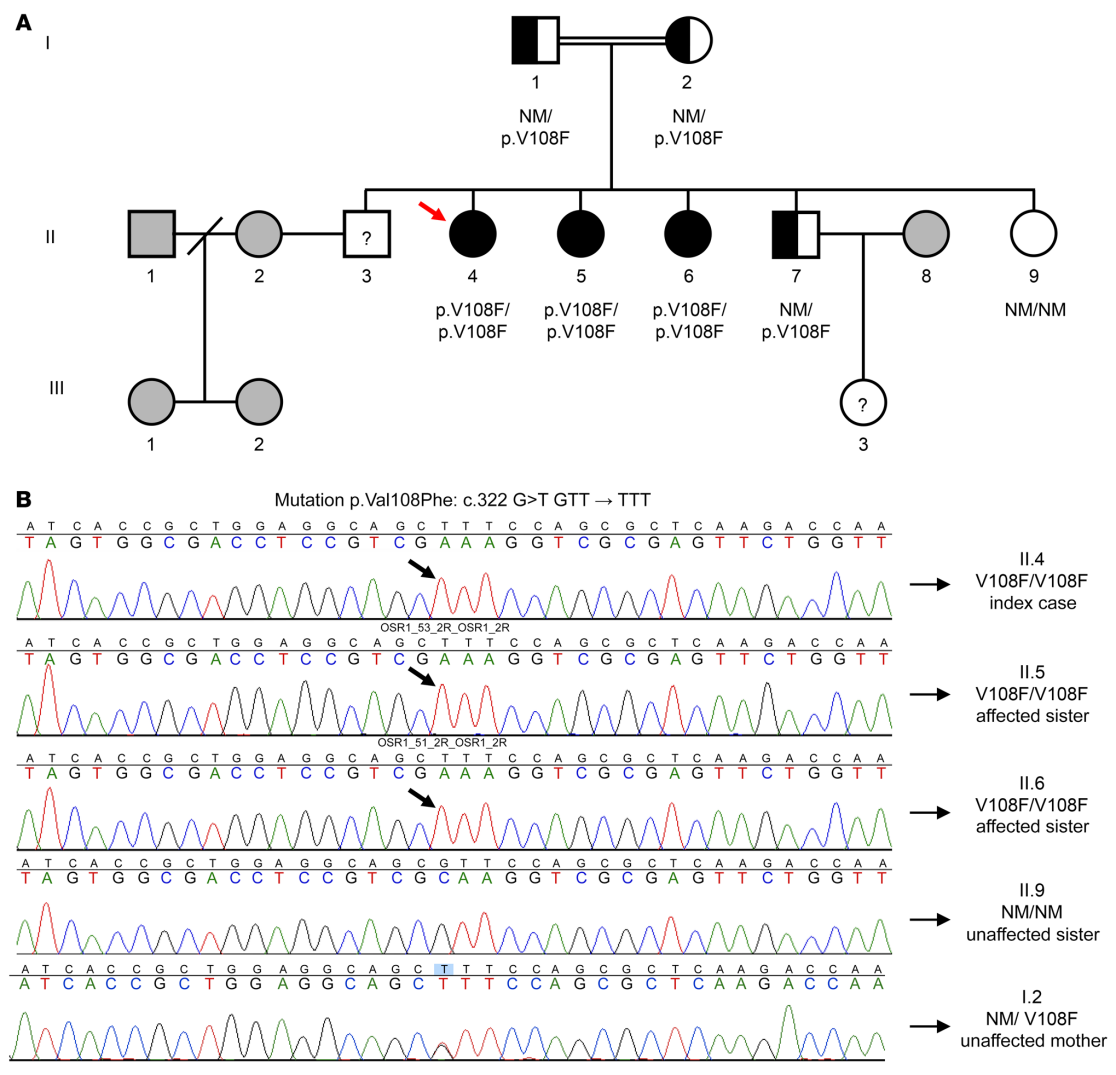
A missense mutation in the *OSR1* gene of a 29-year-old woman (proband) with primary amenorrhea and a developmental anomaly of the reproductive tract. (**A**) Family pedigree; squares indicate male family members, circles female family members. (**B**) Partial sequencing chromatographs of *OSR1* gene. (**A** and **B**) The 3 affected sisters are homozygous (black circles (**A**) and black arrows (**B**)) for a missense mutation in the *OSR1* gene, resulting in a p.V108F substitution. The proband (II.4, red arrow) and her affected sister (II.6) developed spontaneous left tubal pregnancies. Parents (I.1 and I.2) are first-degree cousins and are heterozygous (half-black/half-white square and circle) for the OSR1 p.V108F mutation, as is a fertile brother (II.7). The infertile male sibling (II.3) and the niece (III.3) were not available for clinical and genetic evaluation. Unrelated individuals are represented in gray (not studied). NM denotes nonmutated.

Serial hormone and pelvic ultrasonographic findings of all 3 affected sisters are summarized in Supplemental Table 1. A brother was presumably infertile, based on a history of efforts to conceive over more than 2 years with his wife, who already had 2 daughters from a previous marriage; however, he did not present for medical examination. Two unaffected siblings were evaluated: a fertile brother, and a sister with normal pubertal development, normal uterine appearance on TVUS, and regular menstrual cycles, who had never attempted to become pregnant. The family’s pedigree is shown in Figure 2. A diagnosis of a distinct familial Müllerian anomaly was proposed, characterized by uterine hypoplasia, estrogen-unresponsive thin endometrium, widening of the upper third of the vagina, and spontaneous tubal pregnancies.

#### Genetic studies.

Given the extreme rarity of the phenotype, the consanguinity, and the lack of known genetic causes, we performed WES of DNA samples of the 3 affected sisters and unaffected sister and postulated that this syndrome was due to a homozygous autosomal recessive variant. Therefore, we selected variants from the WES data that were present in homozygosity in all 3 affected sisters but were not homozygous in the unaffected sister (10). We only included variants that were not present in the 1,000 Genomes database phase1 (11), the NHLBI Exome Variant Server (12), nor in other samples sequenced in the same run. Based on this analysis, a missense variant that, to our knowledge, was previously unreported was identified in the *OSR1* gene, resulting in a p.V108F substitution at the protein level. Familial segregation analysis confirmed the autosomal recessive mode of inheritance (Figure 2). Moreover, the p.V108F variant, annotated in Ensembl as rs1369348149, was not found in 50 unrelated Brazilian controls, nor was it present in 141,456 individuals from multiple ancestries in gnomAD (13). Functional annotation prediction scores using SIFT and Polyphen2 were 0.55 and 0.08, which correspond to “tolerated” and “benign”, respectively, while a combined prediction analysis tool (CADD) showed a score of 17.21 (< 30). Overall, the in silico functional prediction suggests that the variant is likely benign (14). Nonetheless, array-CGH excluded the existence of copy number variations (CNVs) associated with the phenotype, thereby strengthening the association of the candidate variant identified in the exome with the phenotype.

We next performed homozygosity mapping using SNPs called from the WES data (15), with the assumption that the affected sisters would share a stretch of homozygosity not found in the unaffected siblings. In total, 42,056 SNPs were used in the homozygosity mapping analysis. Homozygosity mapping yielded a single, highest scoring peak of homozygosity on chromosome 2. The peak extended from chr2:9634856 to chr2:31467351 (hg19 coordinates). The next highest scoring peaks had homozygosity scores that were roughly 70% of the lead peak (Supplemental Figure 1). This homozygosity analysis further supports a recessive condition with the causal variant being found in this region of chromosome 2, where *OSR1* is located.

We next conducted gene prioritization of the 126 RefSeq genes located in the homozygosity peak on chromosome 2, using Endeavour (16). Endeavour prioritizes candidate genes based on their similarity — as calculated from data sources such as functional annotations and gene expression — to known genes involved in a biological process or disease, referred to as a training set. Using a list of 13 genes previously implicated in uterine development as the training set (*LHX1*, *PAX2*, *EMX2*, *WNT4*, *WNT7A*, *WNT5A*, *WNT9B*, *HOXA10*, *HOXA11*, *HOXA13*, *RARG*, *CTNNB1*, and *DLG1*), we prioritized all genes found in the stretch of homozygosity. Among these, *OSR1* ranked very highly (6th out of 126 genes). The 5 genes that ranked ahead of *OSR1* in the peak were *YWHAQ*, *PP1CB*, *ALK*, *NCOA1*, and *PUM2*; however, no nonsense or missense coding variants were found in these 5 genes in the proband or her affected or unaffected sisters. This analysis is independent of the exome variant analysis — but not independent of the homozygosity analysis — and, thus, it is quite striking that one of the highest-ranked genes was also one in which our participants had a missense variant. Altogether, despite the benign functional annotation prediction scores, the genetic analysis strongly supported the potential pathogenicity of the *OSR1* variant in the affected sisters.

#### OSR1 expression in the human uterus.

**Figure 3:**
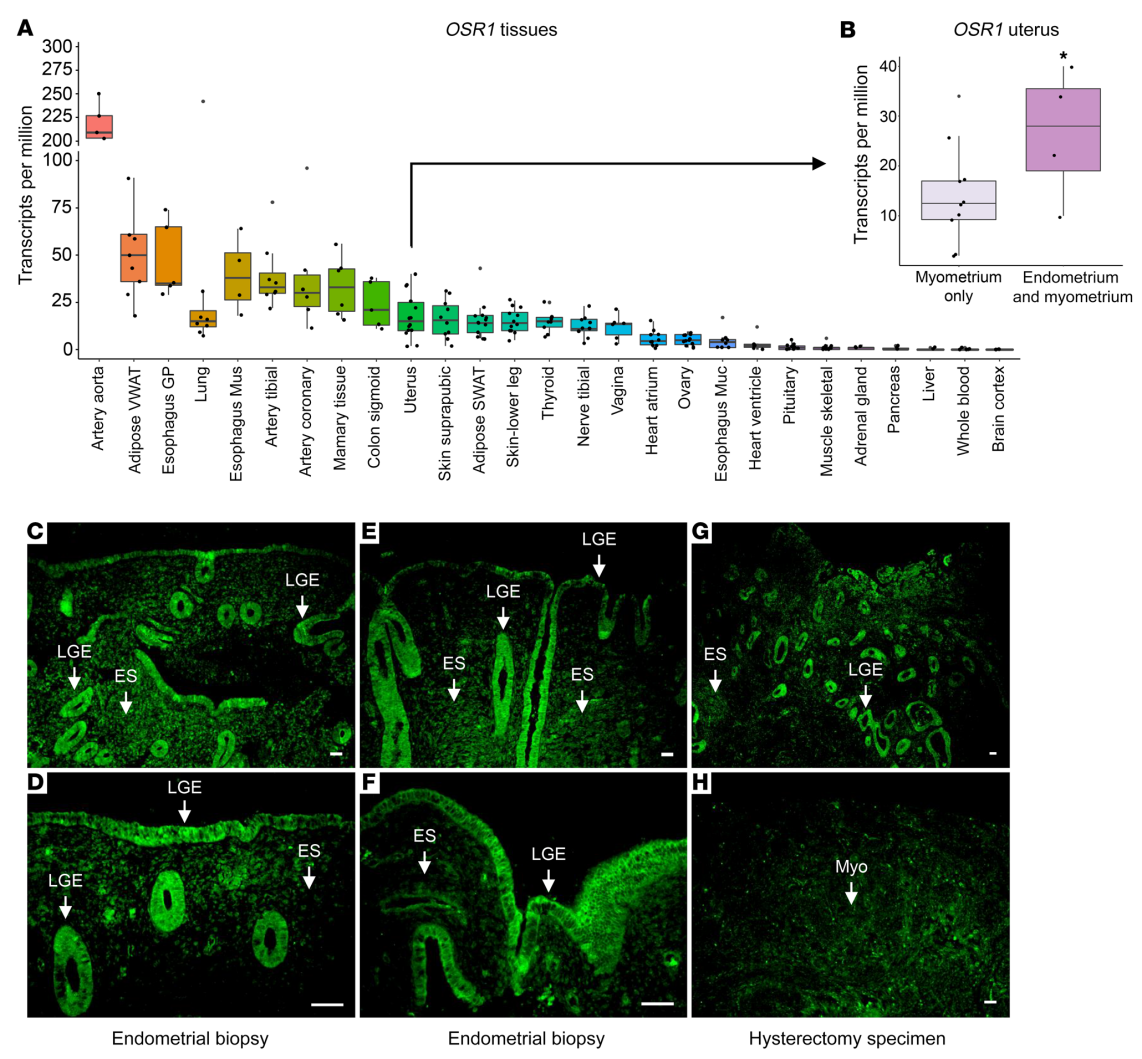
*OSR1*/OSR1 expression in the human uterus. (**A** and **B**) *OSR1* mRNA levels in normal human tissues and uterus. (**A**) *OSR1* mRNA is present at varying levels across a broad range of human tissues, *n* = 4–14/tissue. (**B**) *OSR1* mRNA is enriched in human uterine tissue samples containing both endometrium and myometrium, compared with uterine samples containing only myometrium. Data represent mean ± SEM. *P < 0.05 by 2-sample 1-tailed t test, *n* = 4–10/group. VWAT, visceral white adipose tissue; SWAT, subcutaneous white adipose tissue; GP, gastroesophageal junction; Mus, muscularis; Muc, mucosa. (**C**–**H**) OSR1 protein staining (green) is present primarily in the (**C**–**G**) luminal and glandular epithelial (LGE) cells of the uterus, with lower levels in the endometrial stroma (ES) and in the (**H**) myometrium (Myo) of human endometrial biopsies and uterine samples from hysterectomy. Panels **C** (original magnification, ×10) and **D** (original magnification, ×20) are derived from the same endometrial biopsy sample; panels **E** (original magnification, ×10) and **F** (original magnification, ×20) are derived from the same endometrial biopsy sample. Scale bars: 100 μm.

*OSR1* expression has been demonstrated in many human tissues. However, its expression pattern in the human reproductive tract has not been explored extensively, although OSR1 protein has been detected in the endometrium, fallopian tubes, ovary, and placenta (http://www.proteinatlas.org/ENSG00000143867-OSR1/tissue) (7, 17). We investigated *OSR1* mRNA levels in a variety of normal human tissues and in the postpubertal, developed human uterus. *OSR1* mRNA levels were calculated by summing the number of *OSR1* transcript reads per tissue sample from publicly available Genotype-Tissue Expression (GTEx) Project RNA-Seq data. We found that *OSR1* mRNA is present across a broad range of human tissues, with the highest levels in arteries, adipose tissue, esophagus, lung, mammary tissue, colon, uterus, skin, thyroid, vagina, heart, and ovary (Figure 3A). In the uterus, we found that *OSR1* mRNA is enriched in uterine tissue samples containing endometrium, compared with uterine samples containing only myometrium (Figure 3B). To investigate in greater detail the anatomical distribution of OSR1 in the fully developed human uterus using a validated OSR1 antibody, we localized OSR1 protein in human endometrial biopsies and hysterectomy specimens. In agreement with the transcriptional data, we found that OSR1 protein is present primarily in the luminal and glandular epithelial cells of the uterus, with lower levels in the endometrial stroma and myometrium (Figure 3, C–H). These findings support a role for *OSR1*/OSR1 in the histological patterning of the human uterus, particularly in the endometrium.

### Mouse studies

#### Mouse embryo studies (prenatal development).

*Osr1* is a member of the pair-rule family of genes, characterized by the effects of mutations in them, which impair developmental patterns in alternating segments of the embryo (18). Pair-rule genes play critical roles in embryonic patterning and tissue morphogenesis. Osr1 protein is expressed at a very early embryonic stage in the intermediate mesoderm in the mouse and has been proposed to regulate the differentiation of multiple mesenchymal derived structures, including the formation of the urogenital tract (8, 19).

In keeping with its primordial involvement in organogenesis, previous studies (8) showed that most mouse embryos homozygous for an *Osr1* null mutation (*Osr1*^–/–^) die very early, between E11.5 and E12.5, due to multiple anomalies, including renal agenesis and severe cardiac defects. Moreover, *Osr1*^–/–^ embryos display hypoplastic gonadal ridges and disrupted Wolffian duct (WD) formation at ages E10-E11.5. Interestingly, early WD development was impaired mainly on the left side, and most embryos had truncated or absent WDs on the left side at E11.5 (8).

**Figure 4:**
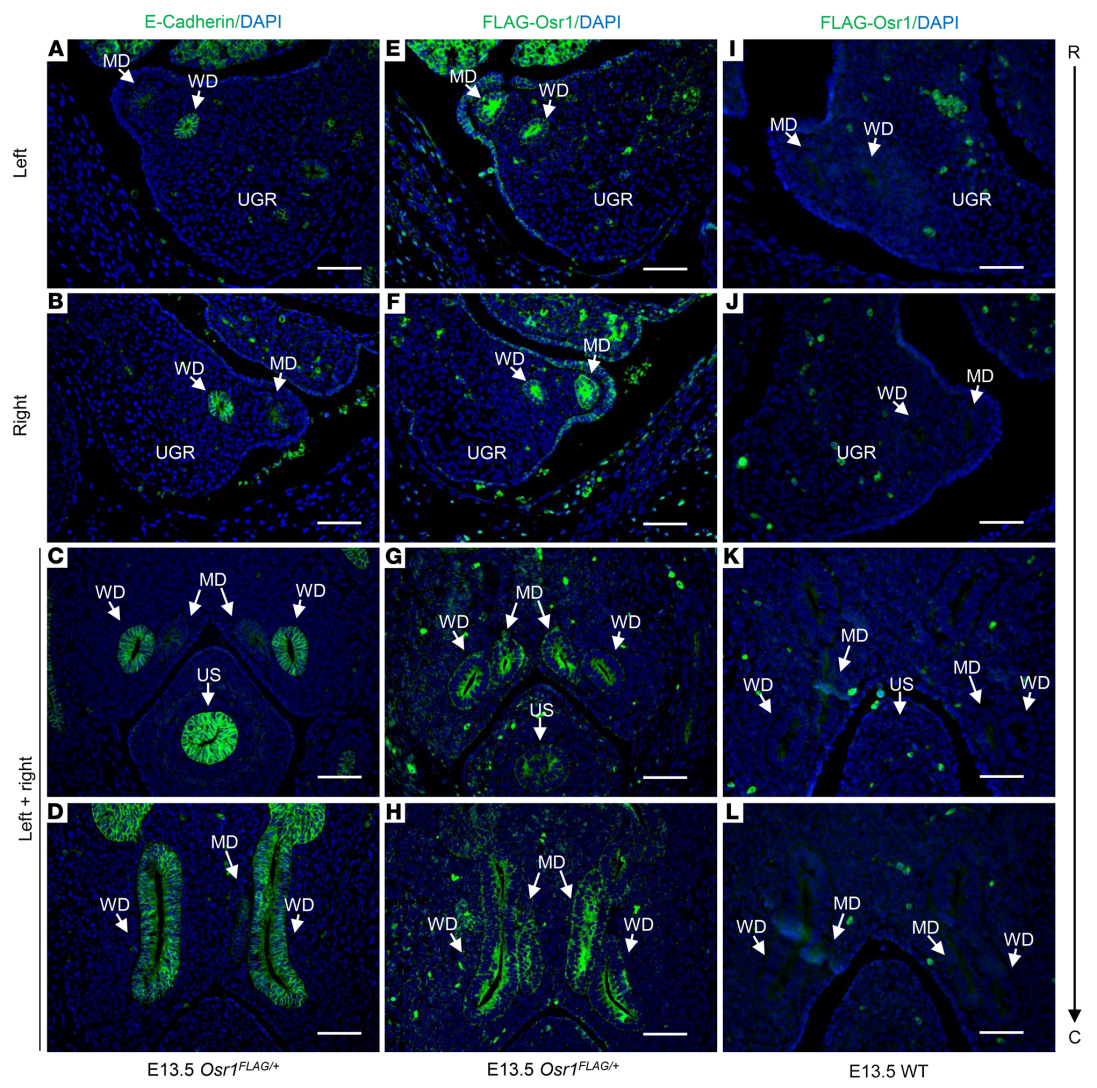
Osr1 expression in the MDs of E13.5 *Osr1^FLAG/+^* mouse embryos. (**A**–**D**) Cross-sections of urogenital ridges showing WDs, distinguished from MDs by staining for E-cadherin (anti-E-cadherin antibody, green), which is an epithelial marker specifically expressed in WDs, but not in MDs, at E13.5. (**E**–**H**) Osr1 is expressed (anti-FLAG antibody, green), in MDs, as well as in WDs, throughout their entire length—i.e., from rostral to caudal segments. (**I**–**L**) Sections of WT E13.5 embryos at the same levels were used as negative controls (anti-FLAG antibody). UGR, urogenital ridge; US, urogenital sinus; R, rostral; C, caudal. Scale bars: 100μm.

Indeed, in mice, MD elongation into the mesonephric mesenchyme is highly dependent on WD development and, specifically, on the secretion of Wnt9b by the surrounding WD epithelial cells (20). Therefore, since WD development is impaired by Osr1 abrogation, we hypothesized that an analogous MD defect would be present in *Osr1*^–/–^ embryos (8). To clarify the effects of Osr1 on MD development, we first assessed the presence of Osr1 protein in the MDs of *Osr1^FLAG/+^* embryos, which express the endogenous Osr1 protein tagged with 2 copies of the FLAG epitope peptide at its amino terminus and had no adverse effect on Osr1 protein function. Using this embryonic mouse model and an anti-FLAG antibody, we found that Osr1 was expressed in the MDs, as well as in the WDs, throughout their entire length — i.e., from rostral segments to caudal terminations in E13.5 embryos (Figure 4). The 2 × FLAG-tagged Osr1 embryos were used for this study because no specific anti-Osr1 antibody was available at the time that this experiment was performed.

**Figure 5:**
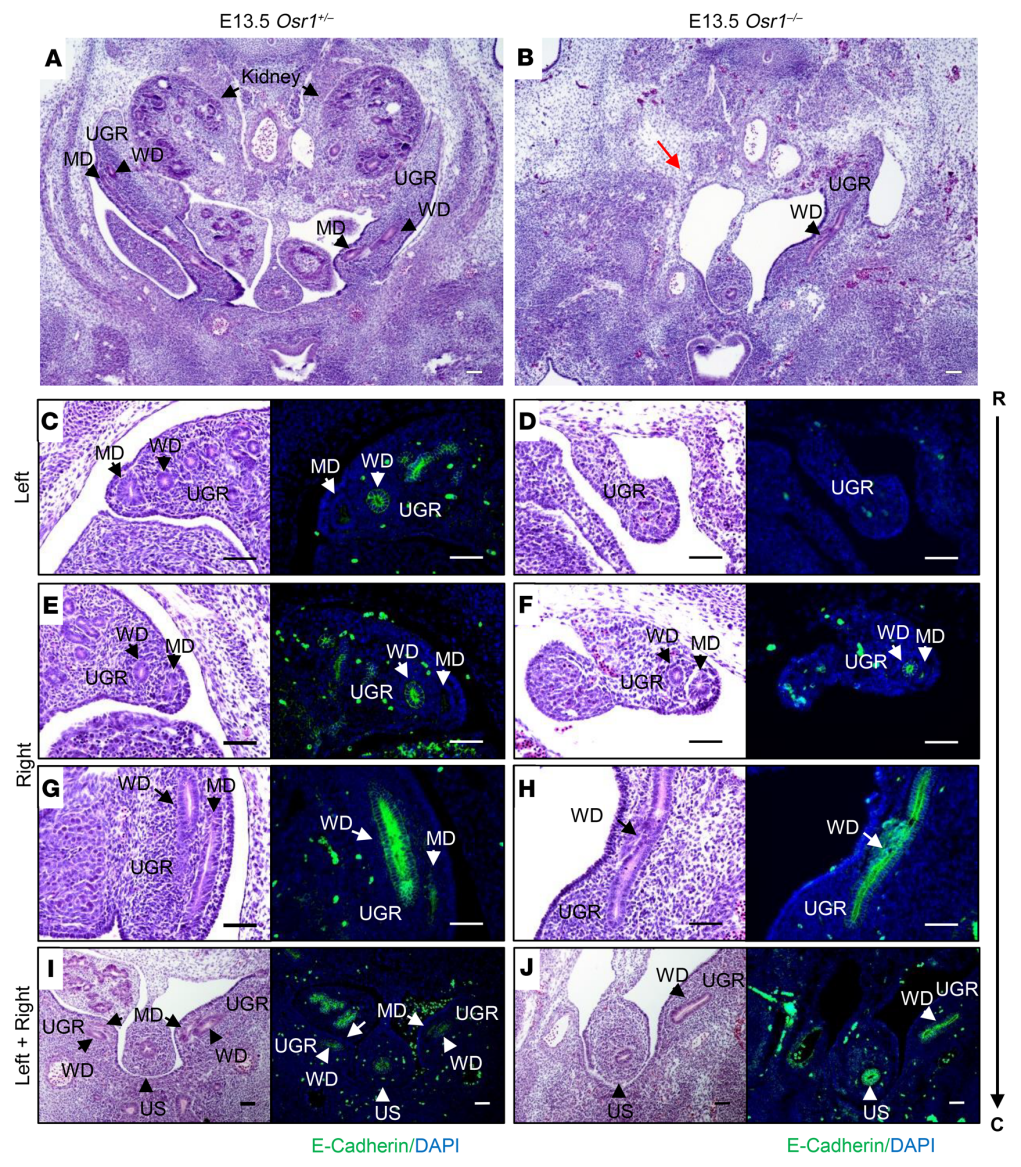
Disruption of MD and WD development in E13.5 *Osr1*^–/–^ embryos. (**A** and **B**) MDs and WDs (black arrows) are both visualized bilaterally in the urogenital ridge (UGR) of an E13.5 *Osr1^+/–^* littermate control (**A**), but both the MDs and WDs are absent on the left side (red arrow) of an E13.5 *Osr1^–/–^* mouse (**B**). (**C**–**J**) H&E-stained cross-sections (columns 1 and 3) extending rostrally to caudally (R → C), with specific E-cadherin immunostaining (green; columns 2 and 4) of the WDs at the same level of the urogenital ridge (UGR), showing that MDs and WDs are absent on the left side (**D**) and MDs are rostrally truncated on the right side (**F**), while WDs appear to have developed normally on the right side of E13.5 *Osr1*^–/–^ mice (**H** and **J**). UGR, urogenital ridge; US, urogenital sinus; R, rostral; C, caudal. Scale bars: 100μm.

**Figure 6:**
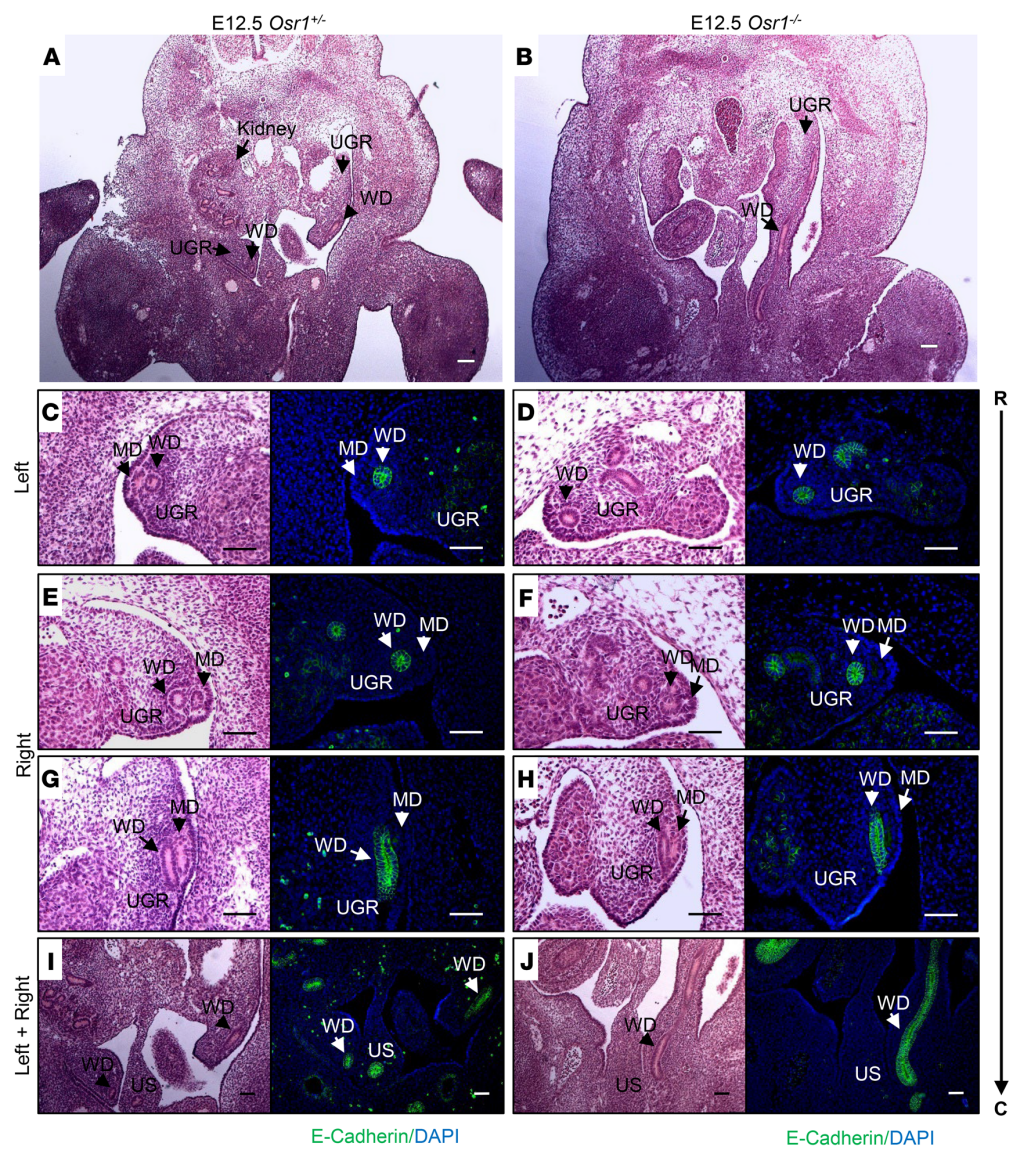
Disruption of MD and WD development in E12.5 *Osr1*^–/–^ embryos. (**A** and **B**) MDs and WDs (black arrows) are both visualized bilaterally in the urogenital ridge (UGR) of an E12.5 *Osr1^+/–^* littermate control (**A**), but the MDs are absent and WDs are truncated on the left side of an E12.5 *Osr1^–/–^* mouse (**B**). (**C**–**J**) H&E-stained sections extending rostrally to caudally (R → C), and specific E-cadherin immunostaining (green) of the WDs at the same level of the urogenital ridge (UGR), showing that MDs are absent and WDs are rostrally truncated on the left side (**D**), and MDs are rostrally truncated on the right side (**F**), while WDs appear to have developed normally on the right side of E12.5 *Osr1*^–/–^ mice (**H** and **J**). UGR, urogenital ridge; US, urogenital sinus; R, rostral, C, caudal. Scale bars: 100μm.

We next analyzed the morphological appearance of MDs in E12.5–E13.5 *Osr1*^–/–^ mice, compared with *Osr1*^+/–^ littermate controls. While the WD was absent on the left side of the E13.5 *Osr1*^–/–^ embryo but preserved on the right side, as previously reported (8), the MD was absent on the left side, and rostrally truncated on the right side as well (Figure 5). WDs were rostrally truncated on the left side at a younger age (E12.5), whereas the morphology of the WDs on the right side was preserved. The rostrally formed MD structure, which is typically seen bilaterally in close contact with WDs in WT E12.5 embryos, was detectable only on the right side of *Osr1*^–/–^ E12.5 embryos (Figure 6). These findings support previous reports that MD development, particularly elongation, is highly dependent on normal WD development (2, 20). However, a direct, cell-autonomous role of Osr1 in MD formation could not be excluded, as MDs did not elongate properly even in the presence of morphologically normal WDs on the right side of the E13.5 *Osr1*^–/–^ embryos (Figure 5).

#### Postnatal development of the mouse FRT.

**Figure 7:**
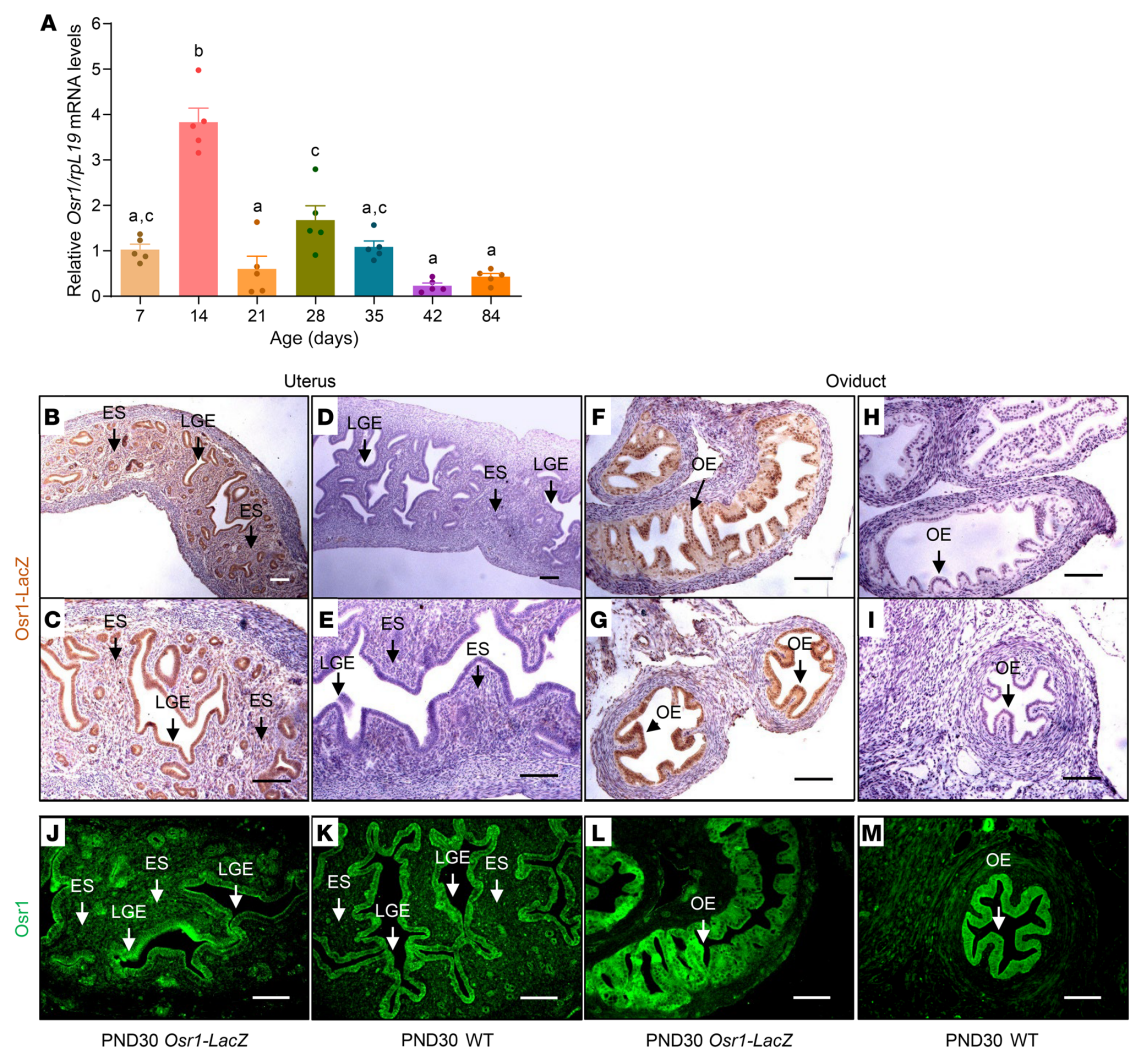
Postnatal expression of *Osr1/*Osr1 in the developing murine FRT. (**A**) *Osr1* mRNA is present across a broad range of ages in the mouse uterus, with the highest levels at PND14, followed by a second, smaller increase from PND28 to PND35. Data represent mean ± SEM. Groups with different letters differ significantly from each other (*P* < 0.05; ANOVA/Tukey) (*n* = 5/group). (**B**–**I**) Osr1-LacZ protein staining (brown) is present primarily in the (**B** and **C**) luminal and glandular epithelial (LGE) cells of the mouse uterus, with lower levels in the in the endometrial stroma (ES) and with high levels in the (**F** and **G**) oviduct epithelium (OE) of PND30 *Osr1-LacZ* female mice, but not in the (**D** and **E**) LGE cells, ES, and in the (**H** and **I**) OE of PND30 *WT* females, confirming specificity of the LacZ staining. (**J**–**M**) Osr1 protein staining (green) is present primarily in the (**J** and **K**) luminal and glandular epithelial (LGE) cells of the mouse uterus, with lower levels in the in the endometrial stroma (ES) and with high levels in the (**L** and **M**) oviduct epithelium (OE) of both PND30 *WT* and *Osr1-LacZ* female mice. **B** (original magnification, ×10) and **C** (original magnification, ×20) are derived from the same uterine sample from a PND30 *Osr1-LacZ* female; **D** (original magnification, ×10) and **C** (original magnification, ×20) are derived from the same uterine sample from a PND30 *WT* female; **F** (original magnification, ×20) and **G** (original magnification, ×20) show different regions of the same oviduct sample from a PND30 *Osr1-LacZ* female; **H** (original magnification, ×20) and **I** (original magnification, ×20) show different regions of the same oviduct sample from a PND30 *WT* female. Scale bars: 100μm.

To further characterize the expression pattern of *Osr1/*Osr1 in the developing FRT, we studied whole uteri of WT C57BL/6 mice across various stages of postnatal development. We found that *Osr1* mRNA is expressed throughout life in the uterus, and that its expression is highest on postnatal day 14 (PND14), compared with the other postnatal ages assessed, followed by a second smaller peak between PND28 and PND35. These findings are noteworthy, given that uterine histoarchitectural development is completed by PND15 in mice, by which time endometrial adenogenesis and mesenchymal specification are complete. Indeed, the highest level of expression of *Osr1* at PND14 coincides with these 2 crucial processes that define the final stages of uterine development in the mouse. It is similarly noteworthy that the secondary *Osr1* expression peak (PND28–PND35) overlaps with the pubertal transition in mice (Figure 7A).

To investigate in greater detail the distribution of Osr1 in the fully developed FRT, we used *Osr1-LacZ* mice at PND30 (8). At this age, the mice are in the early stages of pubertal maturation and a substantial estrogenic effect on endometrial cells is unlikely. Consistent with OSR1 protein expression in the human uterus, we showed that β-galactosidase was present primarily in the luminal and glandular epithelial cells of the mouse uterus in *Osr1-LacZ* female mice at this age, with lower levels in the endometrial stroma and myometrium (Figure 7, B and C), but not in *WT* females (Figure 7, D and E). Additionally, we found that β-galactosidase was present at high levels in the epithelial cells of the mouse oviducts in *Osr1-LacZ* female mice (Figure 7, F and G), but not in *WT* females (Figure 7, H and I). These findings were subsequently confirmed using a validated Osr1 antibody in both WT and *Osr1-LacZ* mice at PND30 (Figure 7, J–M). Therefore, the expression pattern of Osr1 in the postnatal mouse FRT, both at the transcriptional and translational levels, is in keeping with a putative role of this transcription factor in the histological patterning of the FRT, particularly the endometrial and oviduct epithelial compartments.

## Discussion

In the current study, we identified a missense variant in the *OSR1* gene in 3 sisters, born from consanguineous parents, who presented with a unique Müllerian anomaly characterized by uterine hypoplasia with thin endometrium unresponsive to sex steroids, primary amenorrhea, and spontaneous tubal pregnancies. By investigating *OSR1* mRNA levels in a variety of normal human tissues and in the developed, mature human uterus, we found that *OSR1* mRNA is present across a broad range of human tissues, with high levels in the uterus. Additionally, *OSR1* mRNA is enriched in uterine tissue samples containing endometrium, compared with uterine samples only containing myometrium. At the protein level, OSR1 is present primarily in the luminal and glandular epithelial cells of the uterus, with lower levels in the endometrial stroma and myometrium. Using WT and genetically engineered mice, we showed that Osr1 was expressed prenatally in MDs, as well as in WDs, throughout their entire length, from rostral to caudal segments. Moreover, MDs and WDs are absent on the left side and MDs were rostrally truncated on the right side of E13.5 *Osr1*^–/–^ embryos. Postnatally, we found that *Osr1* mRNA was expressed in the mouse uterus throughout the lifespan, peaking at PND14 to PND28. At the protein level, Osr1 is present primarily in the uterine luminal and glandular epithelial cells and in the epithelial cells of the oviducts. Through this translational approach, we demonstrate that *OSR1* is a gene important for MD and endometrial development, and that OSR1 disruption interferes with development of the MD and endometrium, leading to uterine infertility.

In this study, a homozygous *OSR1* mutation was associated with a distinct isolated reproductive tract anomaly. In silico predictions suggested that the OSR1 p.V108F variant had a low likelihood to be deleterious, a finding probably strongly influenced by the fact that this amino acid position is not highly conserved across species, except in primates. Furthermore, despite abundant evidence that Osr1 is a major regulator of the development of other mesoderm derivatives in mice, most notably of the kidney and heart (8), it is intriguing that the 3 women carrying the homozygous p.V108F variant do not have other organ anomalies apart from the identified defects in the reproductive tract. The absence of urological or cardiac abnormalities in the affected women may be related to redundant functions of proteins encoded by odd-skipped genes, or to partial or selective gene inactivation resulting from a single missense mutation rather than a complete gene knockout, which, in mice, leads to severe malformations precluding postnatal survival (8). The human *OSR1* gene encodes a predicted 266-amino acid protein containing 3 C2H2-type zinc fingers and several putative PXXP *src-homology 3* (*SH3*) binding motifs (7), but the OSR1 p.V108F mutation is not located in any of these domains (Supplemental Figure 2). However, recent annotations regarding the variant *OSR1* transcript (rs369348149) indicate that the nucleotide change is located within a regulatory region, a CTCF binding site (14). CTCF is a highly conserved zinc finger protein that can function as a ubiquitous transcription factor. CTCF can also recruit other transcription factors while bound to chromatin domain boundaries, and, thereby, has roles in the regulation of chromatin looping and, consequently, gene expression and genome organization (21, 22). It is therefore plausible that an *OSR1* mutation located in the CTCF binding site would impair gene transcription. Moreover, *NCOA1*, Nuclear Receptor Coactivation 1, is located on the same genomic locus as *OSR1*. NCOA1 acts as a transcriptional coactivator for steroid hormone receptors. It is a member of the SRC family, which has histone acetyltransferase activity and binds nuclear receptors directly, stimulating transcriptional activity in a hormone-dependent fashion. The genomic colocalization of *OSR1* and *NCOA1* together with the finding of uterine estrogen insensitivity in the affected sisters is coincident with a relationship between the OSR1 mutant and impaired endometrial biology in the context of sex steroid dysregulation in the FRT, particularly endometrial estrogen insensitivity and resultant uterine factor infertility.

Despite functional predictions suggesting that the variant p.V108F is likely benign, its extreme rarity — its highest population Minor Allele Frequency (MAF) is less than 0.01 — and the recessive inheritance of the phenotype corroborate a probable causal association. The unique FRT phenotype associated with the previously unreported OSR1 p.V108F variant, together with solid evidence for the importance of OSR1 for mesenchymal development and differentiation, supports the hypothesis that tissue-specific effects of distinct OSR1 variants may occur. Interestingly, a synonymous variant allele of *OSR1*, rs12329305(T), has been associated with reduced nephron numbers and kidney size, as well as increased umbilical cord cystatin C level, in 6% of human newborns, all heterozygotes, from a cohort of 176 healthy-term white infants (17). This variant has a highest population MAF of 0.34 and a CADD score of 0.691, which predict a benign protein functional effect, although in cultured cells it impaired spliceosome binding and mRNA stability (17). This *OSR1^rs12329305(T)^* allele was observed in 15 (12 heterozygotes and 3 homozygotes) of 186 children with high grade, familial and/or bilateral primary vesicoureteric reflux, recruited from Canadian pediatric urology clinics (23). Notably, a small number of *OSR1^rs12329305(T)^* homozygotes have been reported in populations of the Sub-Saharan African region (HapMap), where this allele frequency is higher, but no information regarding kidney anomalies is available (17). In summary, while inactivation of the *Osr1* gene in mice severely compromises metanephric mesenchyme formation, the impact of *OSR1* missense variants on renal development is more variable, and the effects on adult human kidney and urinary tract remain uncertain, with no clinical evidence of a major impact on the nephrogenic phenotype in adult humans to date. Nonetheless, these findings support the notion that *OSR1* missense variants can result in less severe tissue-specific phenotypes than complete abrogation of OSR1 function.

The expression pattern of OSR1 in the human reproductive tract has not been extensively explored. In this study, we showed that *OSR1* mRNA was highly expressed in uterus and enriched in uterine samples in which the endometrium is present, compared to samples with only myometrium. Furthermore, OSR1 protein was detected primarily in the luminal and glandular epithelial cells of the uterus, with lower levels in the endometrial stroma and myometrium. The expression pattern of OSR1/Osr1 in the postnatal human and mouse FRT, both at the transcriptional and translational levels, is in keeping with a putative role of this transcription factor in the histological patterning of the human and mouse FRT, particularly the endometrium.

Osr1 has been shown to be expressed in the mesenchyme and epithelium of the primordial urinary bladder at E10.5 in mice, continuing until birth, with close interaction with key signaling pathways involved in bladder patterning during development (24), analogous to the defects in patterning that we observed in the MDs and WDs throughout embryonic development. In this prior study, the regulatory roles of Osr1 in mesenchymal cell differentiation and collagen production in the urinary bladder were examined postnatally in *Osr1*^+/–^ newborn and adult mice. The authors demonstrated that Osr1 was important for the differentiation of mesenchymal cells that give rise to collagen-producing cells, which enable extracellular matrix deposition in the bladder (24). Similarly, cell signaling between epithelial and mesenchymal components played a role during the process of MD invagination and elongation. It has been proposed that one group of progenitor cells undergoes partial epithelial-to-mesenchymal transition (EMT) in the beginning of MD elongation, while another group undergoes complete EMT and becomes Müllerian mesenchymal cells. Subsequently, partial EMT is reversed and generates differentiated epithelial cells lining the Müllerian lumen. The anterior Müllerian epithelial cells further specialize into the oviduct epithelium. Similar processes remain the basis of cell interplay between surface endometrial epithelium and endometrial stroma in the mature uterus (25). In general, current evidence supports roles for Osr1 in processes of mesenchymal-to-epithelial transition that occur both during embryonic organogenesis and postnatally in mesoderm derivatives. It is thus plausible that a mutant OSR1 protein could affect primarily luminal and glandular endometrial cells.

The isolated reproductive phenotype found in the family reported here, with no detected kidney or urinary tract defects, supports the hypothesis that the functional domain of OSR1 that is mutated in this family may be more selective for WD and MD development in humans. Indeed, it is conceivable that the mutant CTCF binding domain of *OSR1* would impair gene transcription and might impact FRT development selectively, to a greater extent than effects on other tissues. Alternatively, it is possible that other homologous genes, such as *OSR2*, may partially compensate for impaired *OSR1* function during early kidney development in humans, in contrast to mice. Although mouse Osr1 and Osr2 share 98% identity in their zinc-finger (znf) domains and exhibit distinct as well as partially overlapping expression patterns during embryogenesis, targeted null mutations in each of these genes in mice results in distinct phenotypes, with heart and urogenital developmental defects in *Osr1*^–/–^ mice and with cleft palate and open eyelids at birth in *Osr2*^–/–^ mice (8, 19, 26).

It is reasonable to postulate an effect of the p.V108F OSR1 variant on the sequential activation or repression of signaling molecules that underlie the development of Müllerian-derived structures. There is evidence that the *Osr1* gene acts upstream of many transcriptional regulators of intermediate mesoderm cell fate patterning, particularly those involved in kidney development (27). However, direct Osr1 targets have not yet been definitively identified. We demonstrated Osr1 expression in both the WDs and the elongating MDs, and Osr1 ablation, disrupted formation of these structures. These findings support a role for OSR1 in mediating the temporospatial differentiation of the reproductive tract precursors.

Interestingly, some of the developmental genes, such as *Lhx1*, *Pax2*, *Emx2*, *Wnt4*, *Wnt7a*, *Wnt5a*, *Wnt9b*, *Hoxa10*, *Hoxa11*, *Hoxa13*, *Rarg*, *Ctnnb1*, and *Dlg1*, currently known to be involved in urogenital precursor differentiation, are also expressed in adult reproductive tissues, and are modulated by sex steroids (4). These genes play primary roles in the differentiation of the endometrium during the reproductive cycle, decidualization, embryonic implantation, and other postnatal processes that play key roles in fertility (3, 4). It is worth noting that all of the respective human genes (*LHX1*, *PAX2*, *EMX2*, *WNT4*, *WNT7A*, *WNT5A*, *WNT9B*, *HOXA10*, *HOXA11*, *HOXA13*, *RARG*, *CTNNB1*, and *DLG1*), whose functions in urogenital precursor differentiation have been described in mice and that were located in the homozygosity peak in the 3 affected sisters, even though no coding variants were found in these genes, were included in our prioritization analysis, which in turn corroborated *OSR1* as a very likely candidate to be part of this regulatory pathway, based on functional and temporospatial expression analogy. By analogy, our data in mice regarding Osr1 expression in the adult FRT and its embryonic precursors, and the abrogation of MD development in *Osr1^–/–^* mice, parallel the human FRT phenotype associated with the OSR1 p.V108F mutation.

Indeed, most of the current knowledge about MD formation comes from studies of mouse and chicken embryos and involves interactions between developing epithelial and mesenchymal cells, in which several members of the Wnt family of secreted growth factors play determinant roles (4, 28). Among them, Wnt9b is believed to be the main WD-derived signal required for MD elongation, and its targeted deletion results in Müllerian agenesis in mice, while rare human mutations in *WNT9B* have been described in association with the MRKH phenotype (29). Wnt7a, in turn, is considered a marker of MD elongation and is expressed in the MD epithelium during its differentiation and patterning. *Wnt7a*^–/–^ mice have abnormal MD derivatives, characterized by posterior homeotic transformation, in which oviducts are histologically similar to uterus, while uterus transforms to a vagina-like structure (30, 31).

One of the functions of Wnt7a is the maintenance of *Hoxa10* and *Hoxa11* segmental expression along MD development, which is needed for proper patterning (4). Hoxa10 plays important roles during embryonic development of the uterine epithelium, stroma, and muscle (32), whereas its paralog Hoxa11 is primarily involved in the formation of uterine glands and early decidua (33). These transcription factors are characterized by distinct temporospatial expression patterns, which are essential for the determination of the body’s anterior-posterior axis. They are also expressed in the adult endometrium, under regulation by sex steroids, and may participate in embryo implantation, in a process similar to cell fate specification during embryogenesis (32). *Hoxa10*^–/–^ female mice display a disrupted FRT phenotype, in which the uterus is transformed into a narrow tubular structure that resembles an oviduct (34). These mice have impaired embryo implantation in the uterine cavity, although they ovulate and have preserved egg formation. Similarly, abrogation of Hoxa11 leads to implantation defects due to reduced endometrial stromal, glandular, and decidual development in mice (33).

The occurrence of tubal pregnancy in the setting of an estrogen-unresponsive endometrium in 2 of the 3 sisters suggests the existence of an abnormal uterine environment unfavorable for implantation of the embryo, which may be similar in some manner to the disrupted FRT functional patterning observed in *Hoxa10*^–/–^ and *Hoxa11*^–/–^ mice (33, 34), as well as in *Wnt7a^–/–^* mice (30). This unique phenotype is clearly demonstrated by estrogen insensitivity in the uterus. In this context, the genomic localization of *OSR1* in the same locus of *NCOA1* is of note. Notably, the amino acid change of the mutant OSR1 resides within a CTCF binding site. It is thereby tempting to speculate that disrupted interactions between OSR1 and CTCF and/or NCOA1 may underlie impaired actions of sex steroids, notably estrogen, on the reproductive tract of the affected women, particularly the endometrium. Whether estrogen insensitivity also occurs in other segments of the FRT is not yet clear. Estrogen insensitivity throughout the FRT may have also contributed to impairment of oviduct and uterine transport of the sperm and/or egg, but altered endometrial receptivity was likely the main driver of ectopic embryo implantation in these cases. Sex steroid hormone blockade altered ciliary beat frequency in guinea pig fallopian tubes, suggesting a potential mechanism by which estrogen and progesterone impact the tubal transport of embryos (35). Moreover, the impaired hormonal responsiveness of the endometrium in the women with the mutant OSR1 may have compromised endometrial receptivity to embryo implantation, leading to embryo implantation in the Fallopian tube instead of the uterus (36). As endometrial biopsies were not taken in these patients due to the high risk of uterine perforation, endometrial receptivity factors could not be measured.

Lastly, the reproductive phenotype of our proband and her sisters, consistent with endometrial estrogen insensitivity and associated with an OSR1 mutation, is, to our knowledge, previously unreported, as are our findings of defective MD development in Osr1-deleted mouse embryos. Through a translational approach, we demonstrate that *OSR1/Osr1* is a gene important for MD development and endometrial receptivity and is implicated in uterine factor infertility. However, we cannot extend this association to include all cases of thin and E2-unresponsive endometrium. Interestingly, we previously reported absence of endometrium in association with a hypoplastic bicornuate uterus without an OSR1 mutation, further demonstrating that endometrial abnormalities may be seen with defective MD development (37).

Taken together, our results support a role for OSR1, either directly or indirectly through genes regulated by OSR1, in FRT development and in endometrial differentiation during adult life, particularly at the time of embryo implantation. Further studies are needed to investigate the effects of OSR1 on the regulation of sex steroid actions on the FRT of women at reproductive age. In summary, OSR1 is a transcription factor expressed at a very early embryonic stage in the intermediate mesoderm and has been proposed to play critical roles in embryonic patterning and tissue morphogenesis. In addition to the role of OSR1 in the control of intermediate mesoderm differentiation, we propose that the p.V108F OSR1 mutant identified in the homozygous state in our proband and 2 of her sisters may have impaired the coordinated actions of other transcription factors or signaling molecules that regulate MD patterning, with an impact in endometrial differentiation and function across the reproductive life in the affected women. The resulting FRT developmental patterning anomaly and estrogen insensitivity may have impaired embryo implantation in the uterine cavity, thus favoring ectopic pregnancy. The exact mechanisms by which OSR1 may affect the regulatory pathways underlying MD patterning in humans deserve additional investigation. To our knowledge, an association between OSR1 and FRT anomalies has not been described previously. Future studies are necessary to clarify the mechanisms of the action of OSR1 in FRT development and function throughout the lifespan.

## Methods

### Genetic studies

#### WES and familial segregation analysis by conventional Sanger sequencing.

Genomic DNA from all participants was obtained from peripheral blood leukocytes using the salting-out method (38). DNA samples of the 3 affected sisters and 1 unaffected sister were subjected to whole exome sequencing at the Broad Institute (10). Familial segregation of the variant selected based on the WES result was confirmed by PCR amplification and conventional Sanger sequencing. Confirmatory PCR reactions were performed using DNA samples obtained after a second blood collection from the 3 affected and 1 unaffected sister and, additionally, of the mother, father and fertile brother.

#### Array-comparative genomic hybridization.

To investigate whether CNVs could be associated with the phenotype, array-comparative genomic hybridization (aCGH) was performed using DNA samples from all siblings who agreed to participate in this study (39). A 1 × 1 M Agilent SurePrint G3 Human CGH Microarray was performed at the Jackson Laboratory for Genomic Medicine, in which 1 sample was hybridized to 1 full slide with a total of 1 million oligo probes. The slides were scanned using the SureScan Microarray Scanner. Aberration calls were determined by using both the Agilent Cyto Genomics and the Agilent Genomic Workbench analysis software aiming to identify CNVs shared between the parents (or at least 1 of them) and their 3 affected daughters. CNVs that were shared with unaffected siblings were excluded.

#### Homozygosity mapping.

Homozygosity mapping was performed from WES data using HomozygosityMapper (15). To ensure maximum accuracy, we filtered for variants called from WES to include only autosomal SNPs present in dbSNP. We further excluded any variant calls with read depth less than 20 reads. These filters were performed using the Genome Analysis Tool Kit v2.5-2 (40). Genetic homogeneity among affected individuals was required and homozygous stretches longer than 15 SNPs also present in the unaffected sibling were excluded. Block lengths were limited to 250 SNPs and only homozygous blocks longer than 80 SNPs were included.

#### Gene prioritization.

Prioritization analysis of the genes located in the homozygosity peak was performed using Endeavour (https://endeavour.esat.kuleuven.be/) (16, 41). Genes previously implicated in MD development were used as a training set (*LHX1*, *PAX2*, *EMX2*, *WNT4*, *WNT7A*, *WNT5A*, *WNT9B*, *HOXA10*, *HOXA11*, *HOXA13*, *RARG*, *CTNNB1*, and *DLG1*). All RefSeq genes located in the homozygosity mapping peak were used as the candidate gene set. All data sources available in Endeavour were used to build the models.

#### In silico predictions.

The in silico prediction analyses of the potential pathogenicity of the p.V108F OSR1 mutation were performed using SIFT, Polyphen2, and CADD, and corroborated by REVEL, MetalR, and MutationAssessor prediction tools (14).

#### Quantification of OSR1 gene expression in human tissues.

*OSR1* gene expression levels were calculated by summing the *OSR1* transcript reads per tissue sample from publicly available GTEx Project RNA-Seq data. Donors with collected uterine tissue were excluded if: (a) the tissue RNA integrity number (RIN) was less than 6; (b) “slow death” or “ventilator case” was indicated; or (c) uterine histology for the sequenced tissue was not available or GTEx pathologists indicated the presence of cysts or adenomyosis. For samples that met the inclusion criteria, mean expression of *OSR1* was calculated and any samples with *OSR1* expression more than 1 SD from the mean were removed as outliers. Samples were then split into 2 groups based on whether histological observations of the tissue indicated the presence of endometrium and myometrium in the sample or only myometrium. *OSR1* expression was also quantified in every tissue type collected from the donors used in the uterine histology analysis described above. *OSR1* transcript reads were summed to *OSR1* gene level counts in tissues with an RIN greater than or equal to 6. The GTEx uterine sample IDs, tissue donor age brackets, and GTEx pathology notes are described in Supplemental Table 2.

#### OSR1 localization in the human uterus.

Human endometrial biopsies and human uterine samples from hysterectomies, embedded in paraffin (Boston University School of Medicine), were sectioned (7 μm) for IHC. Sections were deparaffinized in xylene, hydrated in 100% and 70% ethanol and submitted to heat-mediated antigen retrieval in 10 mM sodium citrate buffer (pH 6.0) for 20 minutes, at 97°C. Thereafter, tissue slides were blocked in a humid chamber in PBS 1×-Triton X-100-0.3%-NGS (Sigma-Aldrich) 5% for 2 hours at RT. Sections were subsequently incubated in a humid chamber overnight at RT with rabbit polyclonal anti-Osr1/OSR1 (1:500, Thermo Fisher Scientific, PA5-116814) diluted in PBS 1×-Triton X-100-0.3%-NGS 1%. Sections were subsequently incubated with goat anti-rabbit Alexa Fluor 488 secondary antibody (1:1000, Thermo Fisher Scientific, A32731) for 1 hour. Slides were mounted with Vectashield mounting medium with DAPI (Vector lab). The images were captured by using a fluorescent/light microscope. Images were acquired under original magnification, × 4, ×10, or ×20. The rabbit polyclonal anti-Osr1/OSR1 antibody was validated using E12.5 *Osr1*^+/–^ and *Osr1*^–/–^ embryos. Osr1 immunoreactivity (green) was detected in the MDs, WDs, and gonads of *Osr1*^+/–^ embryos (Supplemental Figure 3A), but not in the same structures of the *Osr1*^–/–^ embryos (Supplemental Figure 3B).

### Mouse studies

#### Experimental design and mouse models.

To investigate the expression pattern of *Osr1* mRNA in the developing FRT across different stages of postnatal development, we isolated total RNA from whole uteri of WT C57BL/6 mice on postnatal days 7, 14, 21, 28, 35, 42, and 84. Additionally, to characterize the expression pattern of Osr1 in the MDs, we performed immunolocalization of 2xFLAG-Osr1 protein in the urogenital ridges of *Osr1^FLAG/+^* embryos at age E13.5. The *Osr1^FLAG/+^* mice were generated by CRISPR/Cas9–mediated insertion of the 2× FLAG coding sequence immediately 3′ to the translation initiation codon in the *Osr1* gene, similarly described for the generation of the *Osr1^TY1/+^* mice (42). WT C57BL/6 E13.5 embryo sections were used as negative controls. We also wanted to investigate the effects of Osr1 ablation on MD morphology, therefore, we generated a global knockout mouse model, by serially crossing *Osr1*^fl/fl^ mice (9) with EIIa-Cre transgenic mice, which express Cre-recombinase in the early embryo and cause deletion of loxP-flanked sequences in most tissues, including germ cells. We evaluated MD morphology in H&E-stained sections of urogenital ridges from embryos aged E12.5 and E13.5. WDs were distinguished from MDs by immunofluorescent staining for E-cadherin, an epithelial marker that has been shown to be specifically expressed in WDs but not in MDs at E13.5 (2). Finally, to further determine the localization of Osr1 in the postnatal FRT, we used samples from *Osr1-LacZ* reporter mice (8) at PND30, which were generated by the insertion of a *LacZ* gene into the first coding exon of Osr1 through homologous recombination in ES cells. Sections of uteri from WT mice were used as negative controls.

#### Sample collection and preparation.

Postnatal WT mice were euthanized, and their uteri were collected and stored at –80°C for qPCR analysis. Uteri from PND30 *Osr1-LacZ* females and embryos (at E12.5 or E13.5) from various transgenic mice, as indicated, were dissected, fixed in 4% paraformaldehyde for 24 hours, and then kept in PBS 1× before being embedded in paraffin for histology and IHC.

#### Histology and IHC.

Embryos and uteri were embedded in paraffin (Harvard Medical School Rodent Pathology Core) and sectioned (7 μm) for H&E staining or IHC. All embryos were cross sectioned from caudal to rostral (head down) ends, allowing identification of their right and left sides (Supplemental Figure 4). Images were acquired under original magnification, ×4, ×10, or ×20.

For IHC of FLAG-Osr1, Osr1-LacZ, Osr1, and E-cadherin, uterine and embryo sections were deparaffinized in xylene, hydrated in 100% and 70% ethanol and submitted to heat-mediated antigen retrieval in 10 mM sodium citrate buffer (pH 6.0) for 20 minutes at 97°C. Thereafter, tissue slides were blocked in a humid chamber in PBS 1×-Triton X-100-0.3%-NGS 5% for 2 hours at RT. Sections were subsequently incubated in a humid chamber overnight at 4°C with the specific primary antibodies: mouse monoclonal anti-FLAG, clone M2 (1:200, Sigma-Aldrich, F1804), rabbit polyclonal anti-beta galactosidase (1:500, Thermo Fisher Scientific, 11132), mouse anti-E-cadherin (1:200, BD Biosciences, 610181) or rabbit polyclonal anti-Osr1 (1:500, Thermo Fisher Scientific, PA5-116814) diluted in PBS 1×-Triton X-100-0.3%-NGS 1%. For LacZ-Osr1 staining, slides were incubated with biotinylated goat anti-rabbit IgG secondary antibody (1:1000, Vector lab, BA-1000-1.5) for 1 hour. The reaction product was visualized using 3,3′-diaminobenzidine-DAB Peroxidase (HRP) Substrate Kit (Vector lab). Sections were counterstained with hematoxylin and mounted with mounting medium (Sigma-Aldrich). For Flag-Osr1 and E-cadherin, slides were incubated with goat anti-mouse Alexa Fluor 488 secondary antibody (1:1000, Thermo Fisher Scientific, A32723) for 1 hour. For Osr1, slides were incubated with goat anti-rabbit Alexa Fluor 488 secondary antibody (1:1000, Thermo Fisher Scientific, A32731) for 1 hour. Slides were then mounted with Vectashield mounting medium with DAPI (Vector lab). The images were captured by using a fluorescent/light microscope.

#### RNA extraction and real-time reverse-transcription–PCR.

Total RNA from whole uteri was extracted using TRIzol reagent (Invitrogen), following the manufacturer’s protocol. RNA quantification and purity were assessed using a spectrophotometer (NanoVue Plus, GE Healthcare Life Sciences). RNA was subsequently treated with RNase-free DNase I (Sigma-Aldrich) to avoid genomic DNA contamination. Reverse transcription (RT) and quantitative real time PCR (qPCR) were carried out with 5 ng of total RNA using Power SYBR Green RNA-to-CT 1-Step kit (Applied Biosystems), along with 5 pmol/μL of primers, and were performed in an AB7500 PCR machine (Applied Biosystems). Relative mRNA expression was calculated by the 2^–ΔΔCt^ method using *rpL19* (ribosomal protein L19) as the reference gene (43). Oligonucleotides used for *Osr1* amplification were sense 5′-CTGATGAGCGACCTTACACC-3′ and antisense 5′-TGAGTGTAGCGTCTTGTGG-3′, and for *rpL19,* sense 5′- CTGAAGGTCAAAGGGAATGTG-3′ and antisense 5′- GGACAGAGTCTTGATGATGTC- 3′.

### Statistics

*Osr1* mRNA expression was analyzed using 1-way ANOVA followed by Tukey’s posthoc test performed with GraphPad Prism 5.0 statistical package. *OSR1* mRNA levels were analyzed using 2-sample 1-tailed *t* tests performed with R version 4.2.1. All data sets are presented as mean ± SEM. Statistical significance was considered at *P* < 0.05.

### Study approval

The study was approved by the Ethics Committee on Human Research from the Faculty of Medicine, University of Brasilia, Brasilia-DF, Brazil (approval number: 1.868.615). All participants were studied only after written informed consent was obtained. The paraffin blocks of human endometrial biopsies and uterine samples were obtained from Boston University School of Medicine with approval from Brigham and Women’s Hospital IRB (2005P001440). The mouse studies were approved by the Brigham and Women’s Hospital Standing Committee on the Use of Animals in Research and Teaching in the Brigham and Women’s Hospital Center for Comparative Medicine (approval number: 2016N000447). Mice were maintained at controlled temperature and illumination (12-h light/dark cycle) and fed standard rodent chow and water ad libitum.

### Data availability

The OSR1 p.V108F variant has been annotated in Ensembl under accession number rs1369348149 and dbSNP record (https://www.ncbi.nlm.nih.gov/snp/rs1369348149#variant_details). Supporting data values associated with the main manuscript, including values for all data points shown in graphs and values behind any reported means, are available in the supplemental Supporting data values file.

## Author contributions

ALP and SAP contributed equally to the study and therefore share first authorship. ALP was assigned the first position because she was primarily responsible for the identification and characterization of the inherited clinical condition and the underlying genetics and performed the initial translational experiments, in vitro and early studies with mouse models. SAP designed, continued, and substantially extended the studies using WT and genetically modified mouse embryos as presented herein. SAP additionally designed, performed and analyzed the experiments to determine OSR1 expression in human uterus. ALP, OLM, and PATA provided clinical care to the study’s participants and performed their clinical characterization, as well as familial segregation analysis of the *OSR1* mutation, Sanger sequencing, and analysis of Brazilian control participants; SAP performed and analyzed all mouse experiments with the assistance of ALP and NA, and of MSC, who assisted in the postnatal *Osr1* expression studies using WT mouse uteri. Additionally, SAP designed, performed and analyzed the experiments for OSR1 expression in human uterus, with the assistance of ANF. AD and MHG designed, performed, and analyzed the exome sequencing, homozygosity mapping and gene priorization; ALP and APA assisted in the exome sequencing data analysis and on the gene priorization strategy and analysis. SFO and CL designed, performed and analyzed the array-CG study. HL and RJ designed and developed the genetic mouse models. JCBB performed and analyzed the experiments for *OSR1* mRNA expression in a variety of normal human tissues and in the developed human uterus. WK provided her expertise as a reproductive endocrinology and infertility specialist from the field of obstetrics and gynecology to provide a more detailed description of the clinical features of the affected individuals, and she provided human endometrial/myometrial samples. RSC and UBK mentored ALP, SAP, and NA throughout the study and supervised all generation, collection, and analyses of research data; ALP and SAP analyzed all the research data and wrote the manuscript, which was reviewed and edited by all coauthors.

## Supplementary Material

Supplemental data

Supporting data values

## Version 1 10/17/2023

In-Press Preview

## Version 2 12/01/2023

Electronic publication
