# Supplementary material for: OSR1 disruption contributes to uterine factor infertility via impaired Müllerian duct development and endometrial receptivity
Source: J Clin Invest. 2023 Dec 1;133(23):e161701. doi: 10.1172/JCI161701 (PMC10688984; doi:10.1172/JCI161701)
Supplement: Supplemental data [file jci-133-161701-s166.pdf]

Supplemental Material

Supplemental Table 1. Results of weekly hormone measurements and pelvic ultrasound scans of three sisters with a complex Mullerian anomaly.[1]

	**II.4 (proband)**				**II.5**				**II.6[6]**				**Normal values[2]**
	**Wk 1**	**Wk 2**	**Wk 3**	**Wk 4**	**Wk 1**	**Wk 2**	**Wk 3**	**Wk 4**	**Wk 1**	**Wk 2**	**Wk 3**	**Wk 4**	
**FSH (mIU/mL)**	10.0	7.5	16.5	5.2	9.6	8.6	3.3	12.3	7.9	10.0	7.9	6.1	F: 2.5 - 10.2<br>M: 3.4 - 33.4<br>L: 1.5 - 9.1
**LH (mIU/mL)**	4.9	6.6	**37.6[5]**	4.3	**21.0**	**15.6**	3.9	10.2	3.9	8.0	7.9	**14.9**	F: 1.9 - 12.5<br>M: 8.7 - 76.3<br>L: 0.5 - 16.9
**E2 (pg/mL)**	7.0	22.9	46.8	54.6	87.9	63.7	138.1	53.7	10.1	7.0	35.8	436.3	F: 19.5 - 144.2<br>M: 63.9 - 356.7<br>L: 55.8 - 214.2
**P4 (ng/mL)**	0.2	<0.2	0.7	**8.7**	<0.2	**7.2**	**5.7**	0.6	0.2	<0.2	<0.2	0.3	F: 0.15 - 1.40<br>L: 3.3 - 25.5
**Endometrium[3] (mm)**	1	1	3	3	0	1	0	2.2	0	1	1	2.4	F: 4.0 - 8.0<br>L: 8.0 - 12.0
**Ovarian Follicles[4] (mm)**	10	12	CL	3	12	5	6	6	10	7	10	CL	variable
**Uterus (cm^3)**	13	18	18	18	14	19	15	18	20	20	17	19	nulliparous:<br>28 – 65

[1]Ultrasonographic monitoring was performed weekly, by the same ultra-sonographer, for 4 weeks (Wk 1-4).

[2]Normal values for adult women (chemiluminescent assays). F: Follicular phase; M: Midcycle; L: Luteal phase.

[3]Endometrial thickness. "0" means undetectable. F: Follicular phase; L: Luteal phase.

[4]Maximum diameter of the detected ovarian follicles; CL: corpus luteum.

[5]LH levels in the midcycle ovulatory range and progesterone levels in the luteal range are highlighted in bold.

[6]Data suggest that blood draw was performed during the downswing of the LH surge with corresponding rises in P4 from <0.2 to 0.3 and LH from 7.9 to 14.9. Ovulation may have just occurred, resulting in the identification of a corpus luteum.

Supplemental Table 2. GTEx uterine sample IDs, tissue donor age brackets and GTEx pathology notes.

Tissue Sample ID	Tissue	Age Bracket	GTEx Pathology Notes
GTEX-113JC-2226-SM-5EGJG	Uterus	50-59	2 pieces; atrophic endometrium
GTEX-12WSD-2826-SM-59HKT	Uterus	60-69	2 pieces, all myometrium, no viable endometrium. Prominent vessels, suspect aliqouts from serosal surface
GTEX-12ZZX-2126-SM-5LZVL	Uterus	40-49	2 pieces, myometrium only, no endometrium noted
GTEX-1313W-2826-SM-5P9G1	Uterus	50-59	2 pieces, myometrium only
GTEX-13N11-1126-SM-5KM41	Uterus	50-59	2 pieces; myometrium only in this section
GTEX-14PJM-2826-SM-69LPV	Uterus	50-59	2 pieces; atrophic endometrium and myometrium
GTEX-15ER7-2426-SM-793B9	Uterus	20-29	2 pieces; myometrium only in this section
GTEX-1A32A-2826-SM-72D5S	Uterus	50-59	2 pieces; largely vascularized myometrium with no endometrium in these cuts
GTEX-1A8FM-2326-SM-7MKGC	Uterus	50-59	2 pieces; atrophic endometrium occupies 20% of 1 piece, 10% of other
GTEX-1H1CY-2126-SM-9OSXV	Uterus	60-69	2 pieces; myometrium only; no endometrium
GTEX-1PBJI-2326-SM-E6CP6	Uterus	20-29	2 pieces, myometrium only
GTEX-T2IS-2226-SM-4DM65	Uterus	20-29	2 pieces, ~8mm x 8mm. 2x2mm focus of totaly autolyzed endometrium in one aliquot
GTEX-YJ8O-1126-SM-5P9IS	Uterus	40-49	2 pieces, all myometrium
GTEX-ZAK1-2526-SM-5S2N7	Uterus	50-59	2 pieces, myometrium, no viable endometrium noted

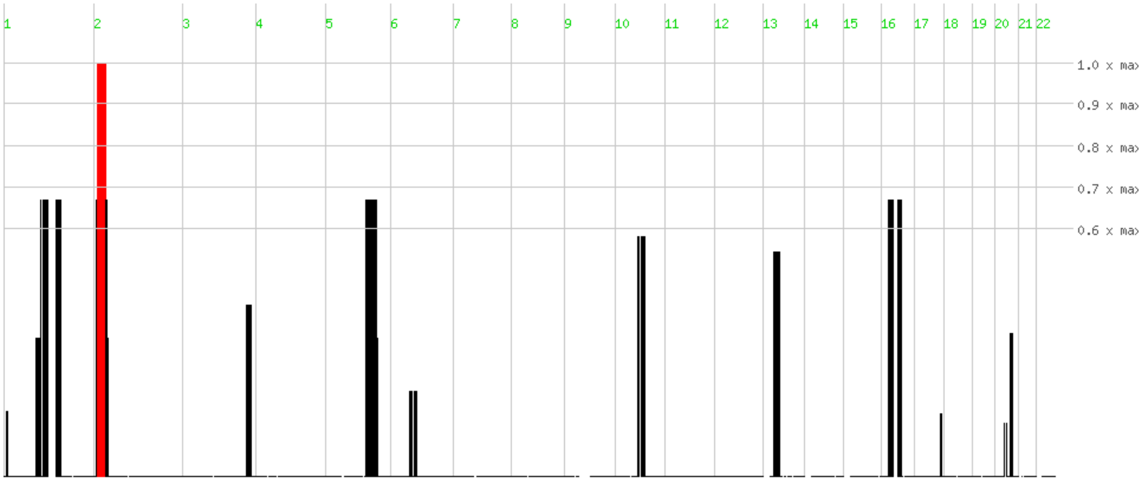

Supplemental Figure 1. Results of homozygosity mapping. Homozygosity mapping was performed from whole exome sequencing data using HomozygosityMapper. The X axis represents chromosomes in which the selected variants that were mapped were located (chromosome numbers are indicated in green across top of chart). Homozygosity scores (normalized to the lead peak, indicated to the right of the chart) are represented on the Y axis. The highest scoring peak is shown in red, while less highly scoring peaks are in black.

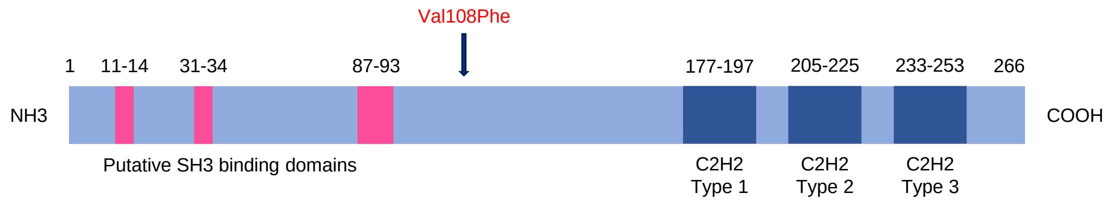

Supplemental Figure 2. Schematic representation of OSR1 domains, with the location of the homozygous mutation (marked in red) identified in three sisters with uterine hypoplasia and estrogen-unresponsive endometrium; two of the sisters also had tubal pregnancies. SH3: Src homology 3; C2H2: Cys_2His_2 zinc finger domain.

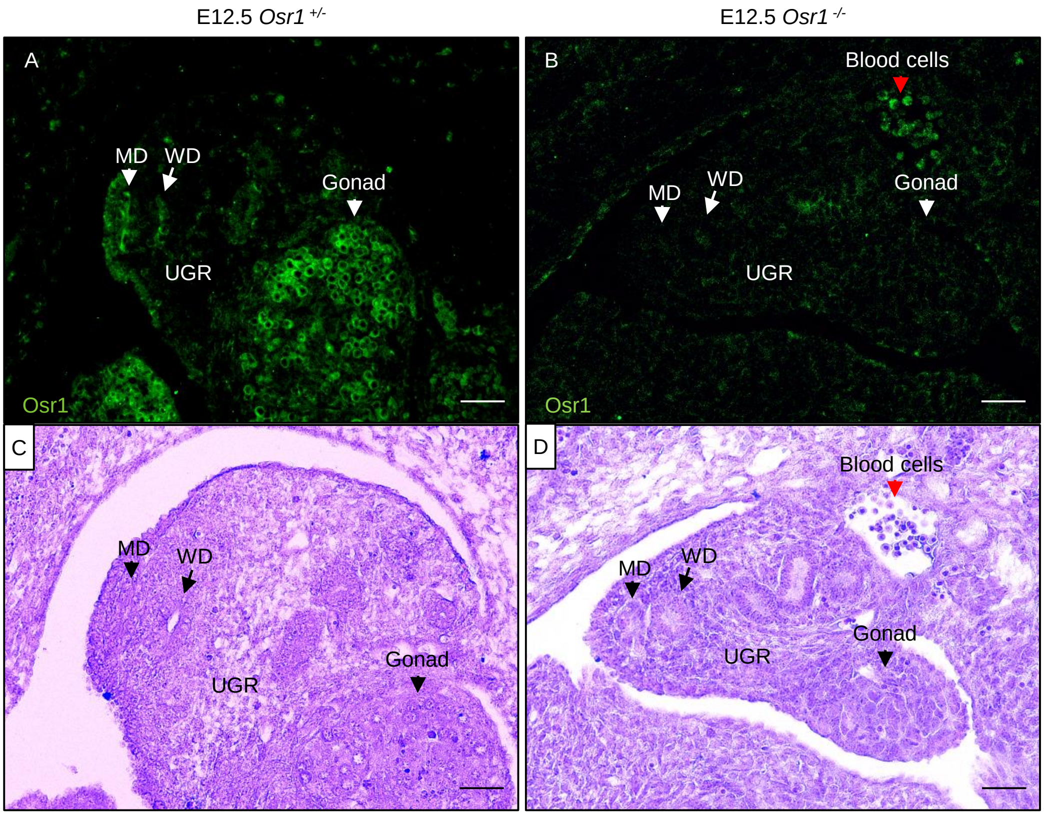

Supplemental Figure 3. Validation of the anti-Osr1/OSR1 antibody. The rabbit polyclonal anti-Osr1/OSR1 antibody (Thermo Fisher Scientific, PA5-116814) was validated using E12.5 *Osr1*$^{+/-}$ and *Osr1*$^{-/-}$ embryos (n=3/group). Osr1 immunoreactivity (*green*) was detected in the MDs, WDs and gonads of *Osr1*$^{+/-}$ embryos (**A**), but not in the same structures of *Osr1*$^{-/-}$ embryos (**B**). Red arrows indicate auto-fluorescent blood cells (*green*). H&E-stained sections of *Osr1*$^{+/-}$ (**C**) and *Osr1*$^{-/-}$ (**D**) embryos. MD, Mullerian duct; WD, Wolf duct; UGR, urogenital ridge; H&E, hematoxylin and eosin stain. Scale bars: 100µm.

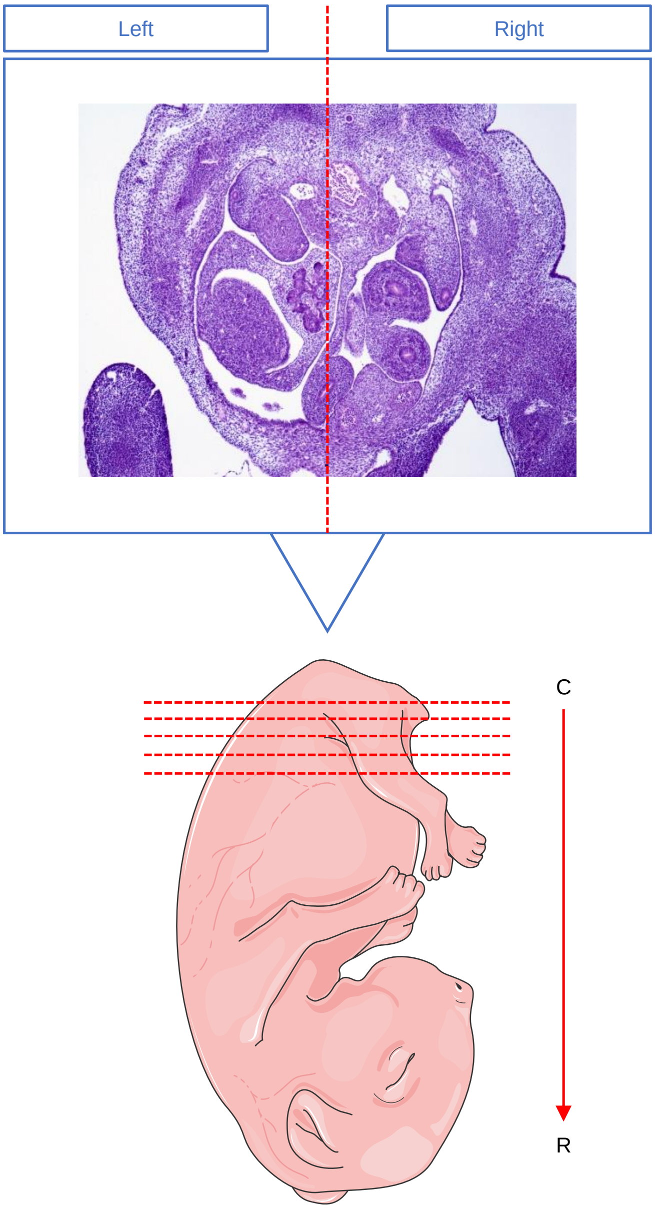

Supplemental Figure 4. Schematic representation of embryo section preparation. All embryos were sectioned from caudal (C) to rostral (R) (head down) allowing the identification of their right and left sides (created from Servier medical arts - https://smart.servier.com).
